#  New Benzodioxole Compounds from the Root Extract of *Astrodaucus persicus*

**Published:** 2016

**Authors:** Saied Goodarzi, Abbas Hadjiakhoondi, Narguess Yassa, Mahnaz Khanavi, Zahra Tofighi

**Affiliations:** Department of Pharmacognosy, Faculty of Pharmacy and Medicinal Plant Research Center, Tehran University of Medical Sciences, Tehran, Iran.

**Keywords:** *Astrodaucus persicus*, Root, Apiaceae, Phytochemistry, Benzodioxole structures

## Abstract

There are many efforts for identification of natural compounds from dietary or medicinal plants. Young roots and aerial parts of *A. persicus* have been used as food additive or salad vegetable in some parts of Iran. In this study, different fractions of the root extract of *A. persicus *were subjected for isolation and purification of secondary metabolites. The methanol extract of the roots was fractionated with hexane (HE), chloroform (CL), ethyl acetate (EA) and methanol (ME). Five novel compounds were isolated from HE, CL and EA using different chromatographic techniques and were identified by ^1^H-NMR,^ 13^C-NMR, 2D-NMR and MS spectroscopic methods. Elucidated compounds with benzodioxole structure were characterized for the first time as 5-((propanoyl methyl)amino)-4,7-dimethoxybenzo[*d*][1,3]dioxole (1), 5-(3-ethyloxiran-2-yloxy)-4,7-dimethoxybenzo[*d*][1,3]dioxole (2), 4,7-dimethoxy-5-(propanonyl) benzo[*d*][1,3]dioxole (3), 4-ethoxybenzo[*d*][1,3]dioxol-6-carbaldehyde (4), and 4-(O-β-D-glucopyranosyl)-6-(3-propanyloxiran-2-yloxy)benzo[*d*][1,3]dioxole (5).

## Introduction

Cancer is a major threatening public health problem and according to a report of the World Health Organization (2007), cancer related deaths are projected to increase up to 18 million in 2020 (1). However, surgery, radiotherapy and adjuvant therapies are still used for treatment of cancer but toxicity, side-effects and resistance against chemotherapy agents necessitates the search for new compounds (2).

Because of the fact that the consumption of fruits, vegetables and herbs lowers the incidence of carcinogenesis, there is a worldwide interest for discovering new natural products or compounds as chemotherapeutic or chemoprotective agents (3). Dietary phytochemicals can induce cellular defense detoxifying/antioxidant enzymes and apoptotic cell death in pre-neoplastic or neoplastic cells through different growth inhibitory mechanisms (4).

The genus of* Astrodaucus* (Apiaceae) is native to different countries from temperate Asia like Iran, Iraq, Syria and Turkey to Eastern Europe like Ukraine (5). *Astrodaucus persicus* (Boiss) Drude and *A. orientalis* (L.) Drude are two species of this genus in Iran (6). Young roots and aerial parts of *Astrodaucus* species have been used as a food additive or salad vegetable in some parts of Iran and Turkey (7). 

The cytotoxicity of the root extract of *A. persicus* was greater than aerial part extract against human breast cancer T47D cells (8). Further investigation on p53 (oncogene), Bcl-2 (tumor suppressor gene) and proteins expressions demonstrated that *A. persicus*, especially its root extract, prevents proliferation of T47D cells by mechanisms such as apoptosis (9). Both aerial part and root extracts of *A. orientalis*, another species of *Astrodaucus* genus, induced apoptosis on T47D cell line but unlike *A. persicus*, the effect of aerial part extract was more prominent than root extract (10). Another study exhibited that *A. orientalis *root extract had phytotoxic activity and strong cytotoxic effects against Mc-Coy cell line with IC_50_ value of 349 µg/mL (7). 

**Figure 1:**
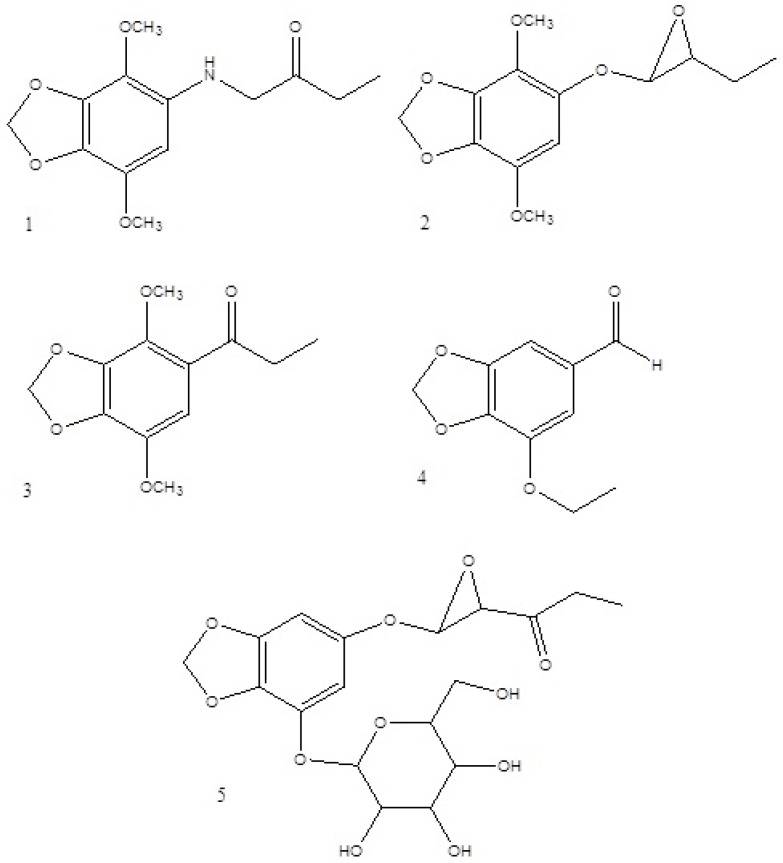
Structure of pure compounds.

Despite of confirmed antiproliferative effects of *Astrodaucus* species, there were few reports about phytochemical constituents. 2, 4-dihydroxyphenyl (E)-6- octadecenoate was the only substance elucidated from n-hexan leaves extract of *A. orientalis *(7). The other investigations were about chemical composition of essential oils of species from various origins (11-14). Thus, the aim of this study was elucidation and identification of natural compounds of *A. persicus* root extract.

## Experimental

*Materials and methods*

*Plant material*

Roots of *Astrodaucus persicus *were collected from Irankhah village, Kordestan Provinces (west of Iran) in September 2012. A voucher specimen of plant is deposited in Herbarium of Institute of Medicinal Plants, Jehad-e-Daneshgahi, Tehran, Iran (Voucher No. 2844 MPIH).

*Extraction and Purification*

The powder of dried roots of *A. persicus *(1190 g) was macerated with 80% methanol at room temperature. The methanol extract was concentrated under reduced pressure yielded 42.5 g crude extract. Crude extract was fractionated with hexane (HE, 15.6 g), chloroform (CL, 7.5 g), ethyl acetate (EA, 2.5 g) and methanol (ME, 15.3 g). The dried fractions were kept at 4 °C prior to test.

*Isolation and purification of compounds*

HE fraction (15.6 g) was loaded on silicagel column chromatography (5.0×31.0 cm) using one step gradient of hexane/ethyl acetate (10:0, 9:1, etc.) to yield 10 subfractions (H_1_-H_10_) on the basis of TLC patterns. Subfractions H_3_ and H_4_ were mixed with each other (H_34_, 2.5 g). It was submitted to silicagel column (2.0×80.0 cm) and hexane/ethyl acetate (9:1) was used as eluent to afford 4 subfractions (H_34a_-H_34d_). H34a (30 mg) was further purified on Sephadex LH**-**20 (0.8×70 cm) eluted with methanol to give compound 1 (9 mg). Subfraction H_6_ (680 mg) was subjected to silicagel column (1.0×70.0 cm) using hexane/ethyl acetate (8:2) as mobile phase to yield 6 subfractions (H_6a_-H_6f_). H_6d_ (12 mg) was pure and named compound 2. Subfraction H_2_ (1 g) was fractionated on silicagel column (1.6×80.0 cm) with hexane/ethyl acetate (8:2) as solvent to give 3 subfractions (H_2a_-H_2c_). H_2a_ (12 mg) was applied to a Sephadex LH-20 column (0.8×70 cm) and eluted with methanol to obtain compound 3.

CL fraction (7.5 g) was submitted to silicagel column (5.0×29.0 cm) and eluted with gradient of chloroform/ethyl acetate (10:0, 9:1, etc.) to obtain 25 subfractions (C_1_-C_25_). Subfraction C_12_ (140 mg) was selected for further separation on another silicagel column (2.0×40 cm) using a step gradient of chloroform/ethyl acetate (19:1, 9:1,7:3), to afford 9 subfractions (C_12a_-C_12i_). C_12c_ (22 mg) was a pure component and named compound 4.

EA fraction (2.5 g) was selected for separation on silicagel column (2.0×29.0 cm) using a step gradient of chloroform/ethyl acetate (5:5, 4:6, etc.), followed by methanol. From 12 yielded subfractions (E_1_-E_12_), E_5 _subfraction was rechromatographed on Sephadex LH-20 column (2.0×95.0 cm) eluted with methanol to afford compound 5 (8 mg).

## Results

*Spectral analysis of compounds*

The isolated compounds were identified using one and two dimensional NMR and MS spectroscopic methods (Figure 1).

*5-((propanoylmethyl)amino)-4,7-dimethoxybenzo[d][1,3]dioxole (1):*

^1^H NMR (400 MHz, DMSO-*d*_6_): δ 6.88 (1H, *s*, H-6), 6.07 (2H, *s*, H-2), 4.34 (1H, *dd*, *J *= 8.0, 5.2 Hz, NH), 3.87 (3H, *s*, OCH3-4), 3.77 (3H, *s*, OCH3-6), 3.17 (2H, *d*, J = 8.0 Hz, H-1′), 2.87 (2H, *q*, *J *= 7.2 Hz, H-3′), 1.02 (3H, *t*, *J *= 7.2 Hz, H-4′); ^13^C NMR (100 MHz, DMSO-*d*_6_): δ 200.2 (*s*, C-2′), 139.9 (*s*, C-3a), 139.0 (*s*, C-7a), 138.8 (*s*, C-7), 138.0 (*s*, C-4), 124.6 (*s*, C-5), 107.9 (*d*, C-6), 102.6 (*t*, C-2), 60.1 (*q*, C- OCH3-7), 56.1 (*q*, C- OCH3-4), 48.6 (*t*, C-1′), 35.8 (*t*, C-3′), 8.32 (*q*, C-4′(; EIMS 70 eV, *m/z* (%): 267 (2) [MW], 253 (4) [MW-CH3+H]^+^, 240 (9.6) [MW-C2H5+H]^+^, 223 (100) [253-OCH3+H]^+^, 195 (13.5) [N-dimethoxybezodioxole], 181 (9.6) [dimethoxybezodioxole], 151 (5.8) [methoxybezodioxole].

*5-(3-ethyloxiran-2-yloxy)-4,7-dimethoxybenzo[d][1,3]dioxole (2):*

^1^H NMR (400 MHz, DMSO-*d*_6_): δ 6.64 (1H, *s*, H-6), 5.98 (2H, *d*, *J *= 0.8 Hz, H-2), 4.95 (1H, *d*, *J *= 4.4 Hz, H-1′), 4.68 (1H, *td*, J = 2.4, 4.4 Hz, H-2′), 3.79 (3H, *s*, OCH3-4), 3.77 (3H, *s*, OCH3-6), 1.53 (2H, *qd*, *J *= 2.4, 7.2 Hz, H-3′), 0.84 (3H, *t*, *J *= 7.2 Hz, H-4′); ^13^C NMR (100 MHz, DMSO-*d*_6_): δ 138.9 (*s*, C-3a), 137.9 (*s*, C-7a), 134.7 (*s*, C-7), 134.5 (*s*, C-4), 131.9 (*s*, C-5), 104.7 (*d*, C-6), 101.2 (*t*, C-2), 101.2 (*d*, C-1′), 67.2 (*d*, C-2′), 59.6 (*q*, C- OCH3-7), 56.5 (*q*, C- OCH3-4), 31.3 (*t*, C-3′), 10.2 (*q*, C-4′(; EIMS 70 eV, *m/z* (%):269 (74.2), 253 (53.2), 240 (48.4), 223 (74.2), 211 (98.4), 181 (25.8), 121 (100).

*4,7-dimethoxy-5-(propanonyl) benzo[d][1,3]dioxole (3):*

^1^H NMR (400 MHz, DMSO-*d*_6_): δ 6.92 (1H, *s*, H-6), 6.17 (2H, *s*, H-2), 3.91 (3H, *s*, OCH3-4), 3.81 (3H, *s*, OCH3-6), 2.91 (2H, *q*, *J *= 8.0 Hz, H-2′), 1.05 (3H, *t*, *J *= 8.0 Hz, H-3′); ^13^C NMR (100 MHz, DMSO-*d*_6_): δ 199.7 (*s*, C-1′), 139.9 (*s*, C-3a), 139.1 (*s*, C-7a), 138.9 (*s*, C-7), 138.0 (*s*, C-4), 124.8 (*s*, C-5), 108.0 (*d*, C-6), 102.6 (*t*, C-2), 60.2 (*q*, C- OCH3-7), 56.3 (*q*, C- OCH3-4), 35.7 (*t*, C-2′), 8.4 (*q*, C-3′).

*4-ethoxybenzo[d][1,3]dioxol-6-carbaldehyde (4):*

^1^H NMR (400 MHz, DMSO-*d*_6_): δ 10.05 (1H, *s*, CHO), 7.15 (1H, *d*, *J *= 1.4 Hz, H-7), 7.04 (1H, *d*, *J *= 1.4 Hz, H-5), 6.07 (2H, *s*, H-2), 2.90 (2H, *q*, *J *= 5.6Hz, H-1′), 1.04 (3H, *t*, *J *= 5.6 Hz, H-2′); ^13^C NMR (100 MHz, DMSO-*d*_6_): δ 198.4 (*s*, C-1′), 148.8 (*s*, C-3a), 140.7 (*s*, C-7a), 138.2 (*s*, C-4), 131.3 (*s*, C-6), 112.2 (*d*, C-5), 101.7 (*d*, C-7), 100.2 (*t*, C-2), 30.9 (*t*, C-1′), 8.3 (*q*, C-2′).

*4-(O-β-D-glucopyranosyl)-6-(3-propanyloxiran-2-yloxy)benzo[d][1,3]dioxole (5):*

^1^H NMR (400 MHz, DMSO-*d*_6_): δ 7.43 (1H, *d*, *J *= 1.4 Hz, H-7), 7.19 (1H, *d*, *J *= 1.4 Hz, H-5), 6.12 (2H, *d*, *J *= 0.8 Hz, H-2), 5.40 (1H, *d*, *J *= 4.8 Hz, H-1′), 5.17 (1H, *bs*, H-2′), 5.07 (1H, *d*, *J *= 8.0 Hz, H-1″), 4.60 (1H, *t*, *J *= 4.0 Hz, H-5″), 3.69 (1H, *dd*, *J *= 12.0, 4.0 Hz, H-6″a), 3.46 (1H, *m*, H-6″b), 3.22-3.37 (2H, *m*, H-3″, 2″), 3.16 (1H, *m*, H-4″), 2.97 (2H, *q*, *J *= 7.2 Hz, H-4′), 1.05 (3H, *t*, *J *= 7.2 Hz, H-5′); ^13^C NMR (100 MHz, DMSO-*d*_6_): δ 198.3 (*s*, C-3′), 149.2 (*s*, C-3a), 140.3 (*s*, C-7a), 139.4 (*s*, C-6), 131.3 (*s*, C-4), 112.5 (*d*, C-5), 102.2 (*d*, C-7, C-1′),101.4 (*t*, C-2), 100.5 (*d*, C-1″), 79.2 (*d*, C-2′), 77.3 (*d*, C-5″), 76.7 (*d*, C-3″), 73.0 (*d*, C-2″), 69.6 (*d*, C-4″), 60.5 (*t*, C-6″), 30.9 (*t*, C-4′), 8.4 (*q*, C-5′).

Structures of compounds were identified by comparison with published data (15-17).

## Discussion

Young roots of *A. persicus *has used as food additive in the west region of Iran. Previous studies demonstrated that both aerial parts and root extracts of *A. persicus *showed strong dose and time dependent antiproliferative effects on T47D breast cancer cell line. The pattern of cell cycle arrest was not like doxorubicin and it was similar to RPMI control (8). Both aerial part and root extracts increased the expression of p53 gene (oncogene). Aerial parts extract increased the Bcl-2 (tumor suppressor gene) expression while root extract of *A. persicus* significantly decreased Bcl-2 expression. Both extracts decreased staining of p53 and Bcl-2 proteins (9).

In this study, there were efforts for isolation, purification and identification of natural components from different fractions of *A. persicus* root as known cytotoxic extract. Five pure compounds were elucidated which all of them were new benzodioxole structures. 

The ^1^H NMR spectra of compounds were characteristic for phenolic compounds with some aliphatic chains. A peak at about δH 7.00 (H-7) suggested the presence of an aromatic proton in compounds 1-3. Compounds 4 and 5 showed two protons in this region which belonged to H-7 and H-5. Two electron withdrawing groups of oxygen, which were attached to the methylene group (C-2), desheilded the H-2 protons at about δH 6.00 ppm. Singlet peaks with integral 3, in the region of 3.7-4.0 ppm belonged to methoxy groups. In HSQC spectrum of compound 1, all of protons and carbons were related to each other except of a hydrogen peak at 4.34 ppm which was not related to any carbon. It was indicated that this proton attached to an electron withdrawing group like nitrogen (NH). The other protons and carbons of compounds were detected in spectra, too. 

There were reports for existence of benzodioxole structures in plants. Safrole (1, 3-benzodioxole, 5-(2-propenyl)) were isolated from sassafras plants and camphor wood. It is a component of oils of sassafras, *Heterotropa* genus, nutmeg, star anise, mace, parsley and cinnamon leaf. Isosafrole (1, 2-methylenedioxy-4-propenylbenzene) occurs naturally in the essential oil of *Cananga odorata*. Myristicin (1-allyl-5-methoxy -3,4-methylenedioxybenzene) is another benzodioxole compound, exist in nutmeg and mace essential oil and other spices of Apiaceae like parsley and dill. Apiol (3, 4-methylenedioxybenzene) is extracted from celery, parsley and *Carum petroselinum*. Dillapiole (4,5-dimethoxy-6-prop-2-enyl-1,3-benzodioxole) an isomer of apiol, is extracted from dill seed and fennel root (18-20).

Benzodioxoles were used as antitumor, antibacterial, antifungal, antiparasitic, antimalaria, antioxidant, pesticides and herbicides (21). There were investigations about carcinogenic and other toxicological effects of compounds with 1, 3-benzodioxole structures like safrole, apiol and myristicin (22-24). Etoposide, teniposide and podophylotoxin are clinical antitumor agents which have the methylenedioxy unit in their structures (25, 26). Other studies reported a variety of anticancer drugs with benzodioxole structures showed excellent bioavailability and low cytotoxicity (27). On the other hand, the 1,3-benzodioxole system is also an integral part of many natural products like sesamol and piperine (28, 29).

The previous investigations revealed the importance of 1, 3-benzodioxole system for antitumor activities (30). Jurd *et al*. by studying structure activity relationship (SAR) of podophyllotoxin and its analogues reported converting the methylenedioxy unit to the corresponding methoxy/hydroxy group dramatically reduced the antitumor activity (25). Another research indicated that the antiproliferative effects of these compounds are not solely due to the existence of reactive benzodioxole ring and some safrole derivatives were not able to inhibit cell growth (30). It is well known that C-5 moiety of safrole is the toxicophore unit by formation of diepoxide specious (butadiene dimmers) and the chain length of alkyl group at this position is important for activity (22, 30). There were aldehyde group as short chain at C-5 position of compound 4 which could diminished cytotoxicity of this compound. Another part of molecule which increases cytotoxicity in benzodioxole structures is epoxy group in the chain. Recent studies showed safrole 2, 3-oxide, an electrophilic metabolite of safrole, induced more potent cytotoxic and genotoxic effects than safrole (30-32). Safrole oxide also showed potent effects on vascular endothelial cells (VECs) which might be promising angiogenesis inhibition (33). Elucidated compounds 2 and 5 of *A. persicus* root extract contained epoxy unit in their chain structure.

## Conclusion

The data of this study was the first report for the existence of benzodioxole structures as abundant components of the roots of* A. persicus* extract. 

## References

[1] Serra AT, Poejo J, Matias AA, Bronze MR, Duarte CMM (2013). Evaluation of Opuntia spp derived products as antiproliferative agents in human colon cancer cell line (HT29). Food Res. Int.

[2] Pan MH, Ho CT (2008). Chemopreventive effects of natural dietary compounds on cancer development. Chem. Soc. Rev.

[3] Reddy L, Odhav B, Bhoola KD (2003). Natural products for cancer prevention: a global perspective. Pharmacol. Ther.

[4] Keum YS, Jeong WS, Kong AN (2004). Chemoprevention by isothiocyanates and their underlying molecular signaling mechanisms. Mutat. Res.

[5] Nazemiyeh H, Razavi SM, Delazar A, Asnaashari S, Khoi NS, Daniali S, Nahar L, Sarker SD (2009). Distribution profile of volatile constituents in different parts of Astrodaucus orientalis (L). Drude. Rec. Nat. Prod.

[6] (1987). Flora Iranica. No. 162. Akademiscbe Druck und verlagsanstalt.

[7] Razavi SM, Imanzadeh G, Dolati S, Nejad-Ebrahimi S, Majrouhi AA, Zahri S, Ghasemian A (2011). Phytochemical prospection and biological activity of Astrodaucus orientalis (L) Drude growing wild in Iran. Pharmacologia.

[8] Abdolmohammadi MH, Fouladdel Sh, Shafiee A, Amin Gh, Ghaffari SM, Azizi E (2008). Anticancer effects and cell cycle analysis on human breast cancer T47D cells treated with extracts of Astrodaucus persicus (Boiss) Drude in comparison to doxorubicin. DARU.

[9] Azizi E, Abdolmohammadi MH, Fouladdel Sh, Shafiee A, Amin Gh, Ghaffari SM (2009). Evaluation of p53 and Bcl-2 genes and proteins expression in human breast cancer T47D cells treated with extracts of Astrodaucus persicus (Boiss) Drude in comparison to Tamoxifen, Immunocytochemistry. DARU.

[10] Abdolmohammadi MH, Fouladdel S, Shafiee A, Amin G, Ghaffari SM, Azizi E (2009). Antiproliferative and apoptotic effect of Astrodaucus orientalis(L) Drude on T47D human breast cancer cell line: Potential mechanisms of action. Afr. J. Biotechnol.

[11] Bazargani YT, Almasirad A, Amin G, Shafiee A (2006). Chemical composition of the essential oils of Astrodaucus persicus (Boiss) Drude root, stem/leaves and flowers/fruits. Flavour Frag. J.

[12] Bigdeli M, Rustaiyan A, Ameri N, Masoudi Sh (2004). Essential Oil of Astrodaucus persicus (Boiss) Drude from Iran. J. Essent. Oil Res.

[13] Mazloomifar H, Bigdeli M, Saber-Tehrani M, Rustaiyan A, Masoudi Sh (2003). Essential oil of Astrodaucus orientalis (L) Drude. J. Essent. Oil Res.

[14] Mirza M, Baher Nik Z, Dini M (2003). Chemical composition of the essential oils of Astrodaucu sorientalis (L) Drude leaves and seeds. Flavour Frag. J.

[15] Andrei GC, Braz-Filho R, Gottlieb OR (1988). Allyl phenols from Ocotea cymbarum. Phytochemistry.

[16] Appendino G, Jakovic J, Bossio E (1998). Structural revision of the parsley sesquiterpenes crispanone and crispane. Phytochemistry.

[17] Benevides PJC, Sartorelli P, Kato MJ (1999). Phenylpropanoids and neolignans from Piper regnellii. Phytochemistry.

[18] Buchanan RL (1978). Toxicity of spices containing methylenedioxy benzene derivatives: A Review. J. Food Safety.

[19] Opdyke DL (1967). Monographs on fragrance raw materials. Food Cosmet. Toxicol.

[20] Hsuuw Y, Chan WH (2015). Apoptotic effects of dillapiole on maturation of mouse oocytes, fertilization and fetal development. Drug Chem. Toxicol.

[21] Gupta SD, Rao GB, Bommaka MK, Raghavendra NM, Aleti S Eco-sustainable synthesis and biological evaluationof 2-phenyl 1,3-benzodioxole derivatives as anticancer, DNA binding and antibacterial agents. Arab. J. Chem. 2014 Aug [cited 2015 Jan 5], 1: [9 screens].

[22] Hsiu-Man L, Po-Tsun K, Chao-Lu H, Jung-Yie K, Ho L, Ding-Yah Y, Ya-Yun L Study of the anti-proliferative activity of 5-substituted 4,7-dimethoxy-1,3-benzodioxole derivatives of SY-1 from Antrodia camphorata on human COLO 205 colon cancer cells. J. Evid. Based Complementary Altern. Med. [450529] 2011 [cited 2015 Jan 5], 1: [8 screens].

[23] Leite ACL, Silva KP, Souza IA, Araujo JM, Brondani DJ (2004). Synthesis, antitumour and antimicrobial activities of new peptidyl derivatives containing the 1,3-benzodioxole system. Eur. J. Med. Chem.

[24] Miller EC, Swanson AB, Phillips DH, Fletcher TL, Liem A, Miller JA (1983). Structure-activity studies of the carcinogenicities in the mouse and rat of some naturally occurring and synthetic alkenylbenzene derivatives related to safrole and estragole. Cancer Res.

[25] Capilla AS, Sanchez I, Caignard DH, Renard P, Pujol MD (2001). Antitumor agents, synthesis and biological evaluation of new compounds related to podophyllotoxin, containing the 2, 3-dihydro-1,4-benzodioxin system. Eur. J. Med. Chem.

[26] Chen GL, Yang L, Rowe TC, Halligan BD, Tewey K, Liu L (1984). Non intercalative antitumor drugs interfere with the breakage-reunion reaction of mammalian DNA topoisomerase II. J. Biol. Chem.

[27] Hai-Hong W, Ke-Ming Q, Hong-En C, Yu-Shun Y, Yin L, Man X, Xiao-Yang Q, Li-Fei B, Hai-Liang Z (2013). Synthesis, molecular docking and evaluation of thiazolyl-pyrazoline derivatives containing benzodioxole as potential anticancer agents. Bioorg. Med. Chem. Lett.

[28] Shenoy RR, Sudheendra AT, Nayak PG, Paul P, Kutty NG, Rao CM (2011). Normal and delayed wound healing is improved by sesamol, an active constituent of Sesamum indicum (L) in albino rats. J. Ethnopharmacol.

[29] Srinivasan K (2007). Black pepper and its pungent principle - Piperine: A review of diverse physiological effects. Crit. Rev. Food Sci. Nutr.

[30] Moreira DRM, Leite ACL, Ferreira PMP, Costa PM, Lotufo LVC, Moraes MO, Brondani DJ, Pessoa CO (2007). Synthesis and antitumour evaluation of peptidyl-like derivatives containing the 1,3-benzodioxole system. Eur. J. Med. Chem.

[31] Li-Ching S, Su-yin C, Ming-Huan L, Wen-Sheng C, Kuen-Yuh W (2012). In vivo formation of N7-guanine DNA adduct by safrole 2, 3-oxide in mice. Toxicol. Lett.

[32] Su-yin C, Pei-yi L, Ming-tsung L, Li-ching S, Wen-sheng C, Hui-fen H, Kuen-Yuh W, Hsiu-ching W (2011). Safrole-2, 3-oxide induces cytotoxic and genotoxic effects in HepG2 cells and in mice. Mutat. Res.

[33] Daimon H, Sawada S, Asakura S, Sagami F (1997). Inhibition of sulfotransferase affecting in-vivo genotoxicity and DNA adducts induced by safrole in rat liver. Teratog. Carcinog. Mutagen.

